# Invasive mechanical ventilation at 36 weeks post-menstrual age, adverse outcomes with a comparison of recent definitions of bronchopulmonary dysplasia

**DOI:** 10.1038/s41372-021-01102-w

**Published:** 2021-05-25

**Authors:** Milenka Cuevas Guaman, Nikou Pishevar, Steven H. Abman, Martin Keszler, William E. Truog, Howard Panitch, Leif D. Nelin

**Affiliations:** 1 Neonatal/Perinatal Medicine, Baylor College of Medicine, Houston, TX USA; 2 Department of Pediatrics, The Ohio State University, Columbus, OH USA; 3 Department of Pediatrics, University of Colorado, Denver, CO USA; 4 Department of Pediatrics, Brown University, Providence, RI USA; 5 Pediatrics, Children’s Mercy-Kansas City, The University of Missouri-Kansas City School of Medicine, Kansas City, MO USA; 6 Department of Pediatrics, The Children’s Hospital of Philadelphia, University of Pennsylvania, Philadelphia, PA USA; 7 Comprehensive Center for BPD, Nationwide Children’s Hospital Columbus, Columbus, OH USA

**Keywords:** Outcomes research, Respiratory tract diseases, Epidemiology

## Abstract

**Objectives:**

To determine whether the need for invasive mechanical ventilation (iMV) at 36 weeks PMA in patients with severe bronchopulmonary dysplasia (sBPD) identifies those patients at highest risk for tracheostomy or gastrostomy, and to compare sBPD with recent definitions of BPD.

**Study design:**

Observational study from Jan 2015 to Sept 2019 using data from the BPD Collaborative Registry.

**Results:**

Five hundred and sixty-four patients with sBPD of whom 24% were on iMV at 36 weeks PMA. Those on iMV had significantly (*p* < 0.0001) increased risk for tracheostomy or gastrostomy. The overall mortality rate was 3% and the risk for mortality was substantially greater in those on iMV than in those on noninvasive support at 36 weeks PMA (RR 13.8, 95% CI 4.3–44.5, *p* < 0.0001). When applying the NICHD definition (2016) 44% had Grade III BPD. When applying the NRN definition, 6% had Grade 1 BPD, 70% had Grade 2 BPD, and 24% had Grade 3 BPD.

**Conclusions:**

Patients with sBPD who were on iMV at 36 weeks had a significantly greater risk of inhospital mortality and survivors had a significantly greater risk of undergoing tracheostomy and/or gastrostomy. The use of type 2 sBPD or Grade 3 BPD would enhance the ability to target future studies to those infants with sBPD at the highest risk of adverse long-term outcomes.

## Introduction

Despite marked improvements in the survival of extremely low birth weight preterm infants, bronchopulmonary dysplasia (BPD) remains the most common cause of morbidity and mortality in this population. In 2000, an NICHD workshop proposed a consensus definition of BPD that has become the most commonly used definition of BPD in the literature for the past 20 years. Using the 2000 consensus definition, severe BPD (sBPD) is defined as the need for positive pressure respiratory support or ≥30% oxygen at 36 weeks post-menstrual age (PMA) [1, 2]. However, many controversies persist regarding definitions of BPD and how to classify disease severity, which have resulted in new proposed definitions [3–5]. Definitions of BPD are especially important for developing standards of care, identifying disease mechanisms, anticipating childhood outcomes, and for designing rational clinical trials for the development of novel therapies [6–9]. Furthermore, the designation of sBPD from the 2000 NICHD workshop encompasses a very wide spectrum of disease severity; including infants requiring low-flow nasal cannula supplemental oxygen at an effective FiO_2_ of 0.30 to infants remaining dependent on invasive mechanical ventilation (iMV) at 36 weeks PMA [1, 2]. Recent studies have suggested that those infants who require higher levels of respiratory support, are especially vulnerable for having adverse outcomes including decreased survival, concurrent neurodevelopmental delays, and other co-morbidities [3, 10, 11]. However, data that more precisely identifies a specific subgroup of sBPD infants at the highest risk of adverse outcomes is lacking.

As part of the BPD Collaborative, we suggested dividing sBPD into two sub-catergories based on the respiratory support that patients were receiving at 36 weeks PMA, where type 1 sBPD would include those patients on nasal cannula or noninvasive positive pressure support (i.e., high flow nasal cannula (HFNC), nasal continuous positive airway pressure (nCPAP), noninvasive intermittent positive pressure ventilation (nIPPV)) and type 2 sBPD would include those infants receiving iMV [12]. We based these 2 sub-categories of sBPD on the postulate that patients with type 2 sBPD would have greater mortality and morbidity than patients with type 1 sBPD. The variables in this study to evaluate morbidity were defined as the need for tracheostomy or gastrostomy, need for respiratory medications during NICU stay and at discharge, repiratiory support at discharge, length of stay (LOS), total ventilator days, and growth velocity ((discharge weight − birth weight)/LOS). Thus, the objective of this study was to determine if patients with sBPD who are receiving iMV at 36 weeks are more likely to suffer adverse outcomes. The BPD Collaborative has recognized the need for a more definite method of identifying disease severity among those patients currently categorized as sBPD [12]. The BPD Collaborative consists of sites interested in the care and outcomes of sBPD and who have established interdisciplinary care programs for the management of patients with sBPD. For this study we used data from the BPD Collaborative Registry. We also examined how the BPD Collaborative modification of the 2000 NIH Consensus Statement definition of sBPD compared with two recently proposed definitions of BPD using this cohort [4, 5].

## Materials/subjects and methods

### Data sources and study sample

There were six centers in the BPD Collaborative that had entered data into the inpatient data registry at the time of this data pull. The hospital are: Children’s Mercy Hospital, Joe DiMaggio Children’s Hospital, Johns Hopkins Hospital, Nationwide Children’s Hospital, Vanderbilt University Medical Center and Women and Infants Hospital of Rhode Island. Each institution contributing data to the registry obtained local IRB approval with waiver of consent. Each institution contributing data also entered into a Data Use Agreement with Nationwide Children’s Hospital where the Registry is housed. The BPD Collaborative Data Registry collects a standardized dataset on all patients born at <32 weeks are entered into the data registry at 36 weeks PMA and were cared for by the dedicated BPD service at their respective institutions. The study was performed in accordance with the Declaration of Helsinki.

The 2000 NIH consensus definition of BPD was used to identify patients with sBPD, and included patients born at <32 weeks gestational age (GA) who received supplemental oxygen for 28 days, and were on ≥30% effective FiO_2_ or positive pressure at 36 weeks PMA [1]. Positive pressure was defined as HFNC, nCPAP, or nIPPV. iMV was defined as receiving IPPV via an endotracheal tube or a tracheostomy. Tracheostomy collar signifies supplemental oxygen delivery to the tracheostomized patient without mechanical ventilation, CPAP/PS (continuous positive airway pressure/pressure support), or CPAP.The data collection included GA and growth parameters at birth and on the day of discharge. Growth velocity was defined as [(discharge weight  −  birth weight)/LOS]. Respiratory support data (FiO_2_ and means of support) were collected at DOL 7, 14, 28, and then at 36 weeks PMA and at discharge along with total ventilator days was collected as well. Data on the use of respiratory medications during the NICU stay and at discharge, such as diuretics, bronchodilators, systemic and inhaled steroids, were collected. Placements of tracheostomies and/or gastrostomies during the hospital stay were recorded. Respiratory support at discharge, classified as follows: none; low-flow nasal cannula (LFNC); tracheal collar; or positive pressure (i.e., HFNC, nCPAP, nIPPV, or IPPV) were recorded.

We also examined how the BPD Collaborative modification of the 2000 NIH Consensus Statement definition of sBPD compared with recently proposed definitions of BPD including the 2016 NICHD workshop definition [5] and the Neonatal Research Network (NRN) definition [4]. The NICHD consensus conference 2016 definition [5] suggested defining BPD in three grades in premature infants born at <32 weeks GA at 36 weeks PMA; where Grade I includes infants on nCPAP, nIPPV, or HFNC (≥3 L/min) at 21% O_2_ or nasal cannula 1 to <3 lpm or hood O_2_ at 22–29%, or nasal cannula <1 L/min at 22–70% O_2_; Grade II includes invasive IPPV at 21% O_2_, nCPAP, nIPPV, or HFNC at 22–29% O_2_, cannula 1 to <3 L/min or hood O_2_ at ≥30% O_2_, and nasal cannula <1 L/min at >70% O_2_; and Grade III includes invasive IPPV >21% O_2_, and nCPAP, nIPPV, or HFNC at ≥30%. The NICHD 2016 consensus definition also included a Grade III(A) which was defined as patients whose early death (between 14 days postnatal age up until 36 weeks) was due to parenchymal lung disease and not associated with any other neonatal co-morbidities [5]. However, we only entered patients into the registry at diagnosis of sBPD at 36 weeks PMA so we were unable to assess earlier deaths. The mortality rate for this cohort was 3% therefore no secondary analysis was done on factors affecting mortality due to the low mortality rate with mortality defined as death prior to discharge with no cutoff for LOS. The NRN definition of BPD [4] was based solely on respiratory support at 36 weeks and included Grade 1 receiving nasal cannula ≤2 L/min; Grade II recieving HFNC, nCPAP, or NIPPV; and Grade 3 receiving invasive IPPV.

### Statistical analysis

The continuous data in this cohort were nonparametric therefore the median and interquartile ranges are shown. The categorical data are shown as number and/or percent. For the nonparametric data comparisons between groups were made using a Mann–Whitney *U* Test or where more than two groups were compared Kruskal–Wallis One Way Analysis of Variance on Ranks was used with a Student Newman–Keuls post hoc test to identify differences between groups. For the categorical data a two-tailed Fisher’s Exact Test was used. Sigmaplot 12.0 (Sigma Scientific, Carlsbad, CA) was used for all statistical tests. A *p* value < 0.05 was considered to denote statistically significant.

## Results

There were 584 patients identified in the Registry with complete data at 36 weeks PMA, of which 20 had moderate BPD by the 2000 NIH consensus definition and were excluded. Of the 564 remaining patients with sBPD by the 2000 NIH consensus definition, 135 (24%) were receiving iMV at 36 weeks PMA. Patients on iMV at 36 weeks PMA weighed less at birth and were born earlier than those sBPD patients on noninvasive support at 36 weeks PMA (Table 1). Patients on iMV at 36 weeks PMA had more total ventilator days, longer LOS, and were treated more often with diuretics, β-agonists, or corticosteroids during their hospitalization than were sBPD patients on noninvasive support at 36 weeks PMA (Tables 1, 3).

**Table 1:** Clinical characteristics of sBPD infants in this cohort.

	Type 1 sBPD	Type 2 sBPD	*p* value
**36 Week PMA respiratory support**			
Number	429 (76%)	135 (24%)	N/A
Birth weight (g)	784 (631–970)	670 (560–831)	<0.0001
Gestational age (weeks)	26 (24–27)	25 (24–26)	0.003
Total ventilator days	24 (7–46)	122 (69–197)	<0.0001
Inhospital mortality	0.7%	9.9%	<0.0001
**Medications during NICU stay**			
Diuretics	49%	78%	<0.0001
β-agonists	39%	72%	<0.0001
Inhaled corticosteroids	46%	70%	<0.0001
Systemic corticosteroids	20%	47%	<0.0001
**Medications at discharge**			
Diuretics	5.5%	14%	0.02
β-agonists	36%	56%	<0.0001
Inhaled corticosteroids	38%	60%	<0.0001
Systemic corticosteroids	1.4%	9.7%	<0.0001
**Procedures during NICU stay**			
Tracheostomy	3.5%	23%	<0.0001
Gastrostomy tube	21%	45%	<0.0001

Continuous data are shown as median (IQR), while categorical data are shown as percentages. The *p* values were calculated using the Fisher’s Exact Test.

**Fig. 1:**
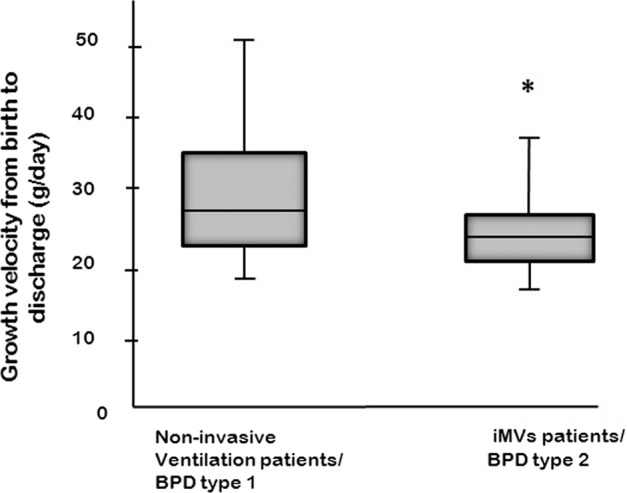
Growth velocity distribution. Growth velocity defined as (discharge weight − birth weight/LOS) is lower in type 2 sBPD than type 1 sBPD. Asterisk indicates (*) type 2 sBPD different from type 1 sBPD, *p* < 0.001. The *p* values were calculated using a Mann–Whitney *U* Test. Invasive mechanical ventilation (iMV).

The overall inhospital mortality rate after 36 weeks PMA for the entire cohort was 3% (16/564). The risk for mortality after 36 weeks PMA was substantially greater in patients receiving iMV at 36 weeks PMA than in those sBPD patients on noninvasive respiratory support at 36 weeks PMA (RR 13.8, 95% CI 4.3–44.5, *p* < 0.0001). The risk for tracheostomy (RR 6.6, 95% CI 3.7–11.5, *p* < 0.0001) or gastrostomy (RR 2.1, 95% CI 1.6–2.8, *p* < 0.0001) were significantly greater for patients receiving iMV at 36 weeks PMA than compared to those who were receiving noninvasive respiratory support at 36 weeks PMA (Table 2). The sBPD patients on iMV at 36 weeks PMA had significantly slower growth velocity than did those on noninvasive support at 36 weeks PMA (Fig. 1).

**Table 2:** Relative risk for adverse outcomes in infants on iMV at 36 weeks PMA.

Variable	RR	95% CI	*p* value
Mortality	13.8	4.3–44.5	<0.0001
Tracheostomy	6.6	3.7–11.5	<0.0001
Gastrostomy tube	2.1	1.6–2.8	<0.0001

The *p* values were calculated using the Fisher’s Exact Test.

**Fig. 2:**
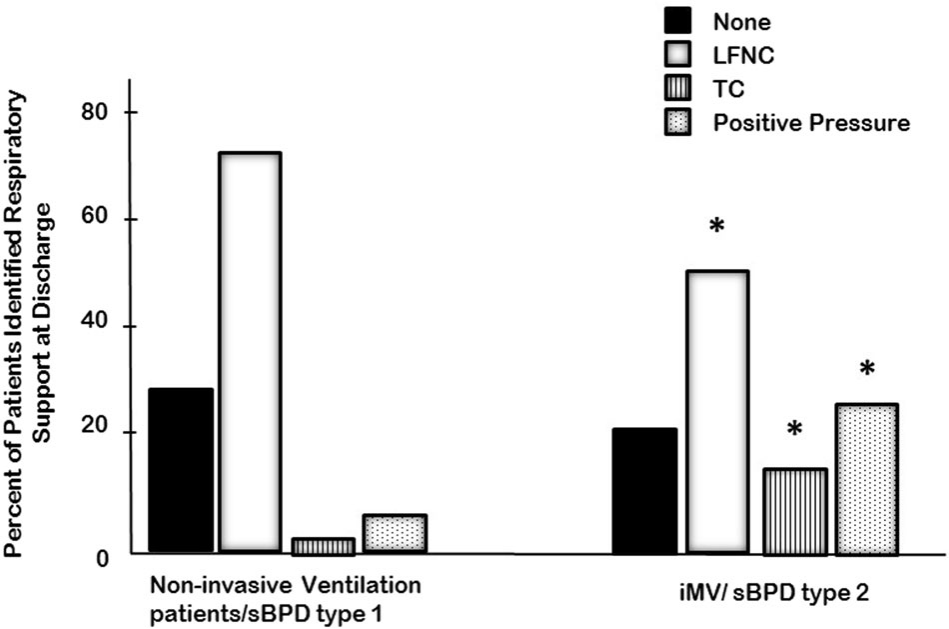
Respiratory support at discharge. Respiratory support at discharge, classified as none; low-flow nasal cannula (LFNC), tracheostomy collar (TC) and positive pressure (i.e., HFNC. nCPAP, NIPPV, or IPPV). Asterisk indicates (*) type 2 sBPD different from type 1 sBPD, *p* < 0.001. The *p* values were calculated using Kruskal–Wallis One Way Analysis of Variance on Ranks with a Student Newman–Keuls post hoc test to identify differences between groups. Invasive mechanical ventilation (iMV), high flow nasal cannula (HFNC), nasal Continuous Positive Airway Pressure (nCPAP), noninvasive intermittent positive pressure ventilation (nIPPV), or intermittent positive pressure ventilation. Tracheostomy collar (TC) signifies supplemental oxygen delivery to the tracheostomized patient without mechanical ventilation, continuous positive airway pressure/ pressure support (CPAP/PS), or CPAP.

Fewer sBPD patients who were on iMV at 36 weeks PMA were discharged on low-flow nasal cannula oxygen compared to sBPD patients on noninvasive respiratory support (Fig. 2). More sBPD patients who were on iMV at 36 weeks PMA were discharged on tracheal collar or positive pressure compared to sBPD patients on noninvasive support at 36 weeks PMA (Fig. 2). The sBPD patients on iMV at 36 weeks PMA were treated more often with β-agonists and corticosteroids at discharge compared to those on noninvasive respiratory support (Table 1).

When applying the NICHD workshop definition from 2016 to our cohort only 94% of the cohort could be classified, and of those that were classifiable 44% had Grade III BPD (Table 3). When applying the NRN definition [10], 100% of the cohort was able to be classified, and of those 6% had Grade 1 BPD, 70% had Grade 2 BPD and 24% had Grade 3 BPD (Table 3). It should be noted that sBPD type 2 and Grade 3 BPD are defined by the same criteria at 36 weeks PMA. Mortality and LOS for the various definitions and severity classifications are shown in Table 3. Only the NICHD definition had patients that could not be classified, and this was 6% of the cohort, and the unclassifiable patients had a mortality of 10% and the longest LOS. The NRN definition placed 6% of patients classified as sBPD into Grade 1 BPD and these patients had no mortality and the shortest LOS.

**Table 3:** Comparison of definitions applied to this cohort.

	Distribution	Mortality	LOS
BPD Collaborative modification of sBPD			
Type 1	76%	0.7%	110 (87–142)
Type 2	24%	9.9%	190 (131–309)
NICHD 2016			
Grade I	26%	0%	95 (69–127)
Grade II	26%	0%	116 (91–149)
Grade III	42%	5.7%	153 (120–214)
Non-classifiable	6%	10%	234 (122–376)
NRN 2019			
Grade 1	6%	0%	99 (86–137)
Grade 2	70%	0.9%	115 (90–146)
Grade 3	24%	9.9%	190 (131–309)

Distribution and Mortality data shown as percent. *LOS* (length of stay) data shown as median (IQR).

## Discussion

BPD was originally described by Northway et al. [13]. Although changes in perinatal and neonatal care over time have improved survival of extremely preterm infants, the incidence of BPD continues to increase [14] and now includes a new phenotype that was characterized as an arrest of lung development by Jobe [15]. The evolving phenotypes of BPD are what lead to the NIH consensus definition in 2000 that included disease severity [1]. There has been growing recognition that the 2000 NIH consensus definition of sBPD includes a very wide distribution of disease severity [3, 10, 16, 17]. This wide distribution of disease severity makes it difficult for clinicians and researchers to determine what sBPD patients are actually at high-risk for mortality and morbidities. As a result, improving the outcomes of patients with sBPD, especially those who remain on iMV for prolonged periods of time, has been difficult within the constraints of the 2000 NIH consensus definition of sBPD. Therefore, we sought to determine if patients with sBPD who remain ventilator dependent at 36 weeks PMA are at the highest risk for adverse outcomes. We chose to assess iMV at 36 weeks PMA because it is an easily identified and objective end-point. Reliance on the FiO_2_ without strict national guidelines for how to wean FiO_2_ results in subjectivity in the definition of BPD and/or the severity grading of BPD. Our findings support the hypothesis that patients who continue to receive iMV at 36 weeks PMA have the greatest risk of mortality and morbidities. Thus, the modification of the 2000 consensus definition of sBPD that we suggested in 2017 with the addition of type 1 and type 2 sBPD does provide a more precise identification of the highest risk infants. We should point out that the NRN definition of Grade 3 BPD also identifies the patients in this cohort with the greatest risk of adverse outcomes. Thus, the use of Grade 3 BPD identifies patients at the highest risk for adverse outcomes and will be useful for future epidemiologic studies and randomized clinical trials.

The lack of consensus in approach to patients with sBPD continues to hamper the development of new methods or therapies to improve outcomes [18, 19]. Large multicenter interventional trials are desperately needed for this patient population, but the lack of consensus introduces the concept of “equipoise”, which then often becomes a reason not to perform the study. Furthermore, the wide spectrum of disease currently classified as sBPD leads to potentially targeting a population that may not be expected to benefit from the therapy. An improved ability to identify the highest risk patients with sBPD will allow for the development and study of precision management strategies tailored specifically to this high-risk group. For example, the highest risk sBPD patients may be those most likely to benefit from pulmonary targeted pharmacotherapies. However, the widely variable phenotype currently lumped under sBPD likely hampers the recognition of meaningful clinical responses to common pharmacotherapies. Most of which are already utilized in this population with little or no evidence to support their use, such as β-agonists, diuretics, and inhaled corticosteroids. It has been found that the utilization of these pharmacotherapies vary widely between centers [18, 19]. In a recent retrospective cohort study using the Pediatric Health Information System database, Bamat et al. [19] concluded that infants with sBPD are exposed to an alarming number of medications of unclear efficacy and safety, with marked variation between centers.

We have utilized the terms type 1 and type 2 sBPD as a method of separating sBPD patients into two distinct potential phenotypes [12]. The definition of sBPD is based on the 2000 NIH consensus definition [1]. However, it is clear 20 years later that the 2000 NIH Consensus definition no longer adequately meets the changing needs of neonatology [10, 20]. In fact, there has been a very lively debate in the literature over the last several years regarding new definitions, what elements they should include, and what they should be able to prognosticate [20–27]. We demonstrate in this study that those BPD patients on iMV at 36 weeks PMA are at the highest risk for inhospital mortality and morbidities. The 2016 NICHD definition of BPD does include newer modes of noninvasive ventilation that weren’t available in 2000, although the definition proposed is based on a very complex interaction between FiO_2_ and respiratory support at 36 weeks PMA [5]. Interestingly, in our cohort 6% of patients were not able to be classified using the 2016 NICHD definition due to lack of reliable FiO_2_ data at 36 weeks PMA. In 2019 the NRN also proposed a series of definitions of BPD based solely on the level of respiratory support needed at 36 weeks PMA and tested the various definitions to find the one that “best” predicted death or serious respiratory morbidity through 18–26 months corrected age using the area under the receiver operator characteristic curve (AUROC) [4]. They found that defining Grade 1 as low-flow nasal cannula, Grade 2 as noninvasive positive pressure, and Grade 3 as iMV gave the best predictive value in terms of the AUROC for late death or serious respiratory morbidity [10]. Although, it should be pointed out that the AUROC was only 0.79, suggesting that either newer definitions and/or developing phenotypic clusters within current definitions are needed to improve the ability to develop a classification scheme that identifies those at the very highest risk for later adverse pulmonary outcomes. The 2019 NRN definition is the easiest to assign since it only requires one timepoint and does not use supplemental oxygen [4]. When the NRN criteria was applied to our cohort, we also found that 6% of our sBPD patients were defined as having Grade 1 BPD, and that the patients identified as Grade 1 BPD had no inhospital mortality and a short LOS. Thus, the NRN criteria may provide a more precise classification compared to our suggested sub-grouping of sBPD since it includes both lower and higher severity of disease.

There are some limitations to our study that should be considered. First, the cohort only consisted of patients with established sBPD as this was our population of interest for this study. However, this may limit the generalizability of our findings to all preterm infants classified as having BPD. Second, it is possible that there is referral bias in our cohort, since the six centers contributing data to this cohort are all centers that have an established BPD program and therefore may not be representative of the entire population of sBPD patients. Finally, our dataset was limited to inhospital mortality and morbidity. Going forward the BPD Collaborative is developing and implementing an outpatient dataset, which will be useful in determining the effect of invasive IPPV at 36 weeks on long-term outcomes.

In conclusion, we found that in a cohort of patients with sBPD, those that were receiving iMV at 36 weeks PMA were at the highest risk of inhospital mortality and morbidity. Patients with sBPD who were on iMV at 36 weeks had a significantly greater risk of inhospital mortality and survivors had a significantly greater risk of undergoing tracheostomy and/or gastrostomy placement prior to discharge. Our study adds support to the growing perception that new definitions and/or classifications of BPD are needed going forward to better capture the breadth of phenotypes and endotypes that characterize this chronic lung disease of prematurity. We suggest that future definitions include objective methods for differentiating the wide spectrum of disease presentation and progression representing the severest forms of BPD. Throughout this study, it has been shown that the BPD Collaborative definition of type 2 sBPD and the NRN definition of Grade 3 BPD are the same diagnosis since it specifies a sub-category of patients who have iMV at 36 weeks PMA, however the NRN definition also categorizes a subset of sBPD infants into a less severe category as well in which those infants had no mortality and a shorter LOS. As a start, the utility of using the NRN definition for Grade 3 BPD would be most feasible given the singular timepoint to make the diagnosis and this would enhance the ability to target future studies to those infants with sBPD at the highest risk of adverse long-term outcomes.

## Data Availability

The BPD Collaborative Data Registry collects a standardized dataset on all patients born at <32 weeks who were cared for by the dedicated BPD service at their respective institutions.
